# Microstructure Evolution and Improved Permeability of Ceramic Waste-Based Bricks

**DOI:** 10.3390/ma15031130

**Published:** 2022-01-31

**Authors:** Wenfei Zhou, Huiling Du, Le Kang, Xian Du, Yupu Shi, Xiaojing Qiang, Haodong Li, Jing Zhao

**Affiliations:** College of Materials Science and Engineering, Xi’an University of Science and Technology, Xi’an 710054, China; zwf137260097@163.com (W.Z.); kangle20140805@126.com (L.K.); cherrydu33@163.com (X.D.); forlisser@xust.edu.cn (Y.S.); qxjjj1204@163.com (X.Q.); LI15902950637@163.com (H.L.); ZhaoJ1016@163.com (J.Z.)

**Keywords:** waste ceramic materials, magnesium slag, coal gangue, sintered brick, permeability

## Abstract

The resource and large-scale utilization of waste ceramic materials, magnesium slag, and coal gangue are one of the important ways for the sustainable development in metallurgy, coal, and other related enterprises. In this paper, waste ceramic materials were used as aggregates; coal gangue and magnesium slag were used as mixed binder; and the all solid-waste-based permeable bricks with excellent performance were prepared by forming pressure at 5 MPa. The mechanical properties and water permeability of the all-solid-waste-based permeable bricks were evaluated. The results proved that the porous channel of permeable brick is mainly composed of waste ceramic materials with a particle size of 2–3 mm. Pore structures below 200 μm were mainly composed of fine aggregate and mixed binder. Using 60% coarse aggregate, 20% fine aggregate, 10% coal gangue, and 10% magnesium slag as raw materials, the all-solid-waste-based permeable bricks were obtained by pressing at 6 MPa and sintering at 1200 °C, which exhibited the best performance, and its water permeability, compressive strength, and apparent porosity were 1.56 × 10^−2^ cm/s, 35.45 MPa, and 13.15%, respectively. Excellent water permeability, compressive strength, and apparent porosity of the all solid-waste-based permeable bricks were ascribed to the high content of connecting open pores, and closely adhesive force were ascribed to the porous microstructure constructed by the grading of waste ceramic materials and the tight conjoined points of the liquid phases in coal gangue and magnesium slag at a high sintering temperature.

## 1. Introduction

Rapid developments of industrialization are acquired owing to the use of massive amounts of minerals and fossil resources [1,2]. Considerable industrial solid wastes (ISWs) are piling up everyday, leading to extreme pollution in air and water conditions, posing unexpected dangers to the environment [3,4,5]. Therefore, ISW must be managed in appropriate and low energy-consuming ways. Nevertheless, ISW is a so-called misallocation of resources [6,7,8]. Utilizing and recycling ISW has aroused attention all over the world in the recent decades. As reported by Wang et al. [9], the ceramic industry in China has constantly occupied the first place in the world, while about 30% of production in the ceramic industry goes to waste [10]. Waste ceramic materials (CW), which are difficult to degrade because of their durable and highly resistant characteristics [11], have been used in civil engineering and, more specifically, in calcined cement clinker, active admixtures, and cementing materials [12]. However, its use rate is still limited. Meanwhile, coal gangue (CG), a by-product in the coal industry, has been widely studied and used in studies [13,14,15,16]. Currently, many researchers have focused on studying the use of CG in pavements and cementing building materials thanks to its high content of Al_2_O_3_ and SiO_2_. However, the annual utilization of CG in China is only 60% [17]. By contrast, the singleton of magnesium metal can produce approximately 5.5–10 tons of magnesium slag [18], which is directly disposed of on cultivated land, causing the presentation of secondary hazards such as atmospheric pollution and soil alkalization [19,20]. The use of MS as an admixture to produce cement is discontinued today. Consequently, the related research on construction materials using MS is rare, even though has a high content of CaO.

Currently, the phenomena of urban heat island effect and urban inland inundation are occasionally presented [11], leading to considerable problems, including but not limited to the cost of property damage. The permeable brick contained various connected open channels, relieving the urban heat island effect and building sponge city [21,22]. Some efforts have been made to use ISW as aggregates and mixing binders in permeable bricks. Yang et al. [10] reused waste ceramic materials prepared for the high permeability and mechanical properties of permeable bricks. Xu et al. [23] sintered the coal gangue and brick at 900–1250 °C, obtaining bricks with high mechanical properties. However, few researchers report and use MS in building materials. This may be because the high content of CaO had a negative impact on mechanical properties. The fundamental properties of permeable bricks are mechanical property and permeability coefficient. However, various reports indicate [24,25] that there is a negative correlation between them. Therefore, the related studies on the application of CG in porous materials are rare owing to its vitrification at high temperatures. To solve the vast accumulation of waste ceramic materials, magnesium slag, and coal gangue, it is necessary to combine those industrial solid wastes with permeable bricks.

In this paper, the novel all solid-waste-based permeable bricks with waste ceramic materials, coal gangue, and magnesium slag were prepared via the pressing-sintering process. The purposes of the research are as follows: (1) prepare the new permeable bricks and achieve the resource comprehensive utilization of waste ceramic materials, coal gangue, and magnesium slag; (2) the prepared permeable bricks meeting national standards provide a new possibility for the selection of pavement materials, so as to realize the large-scale and engineering application of solid waste.

## 2. Experimental

**Figure 1:**
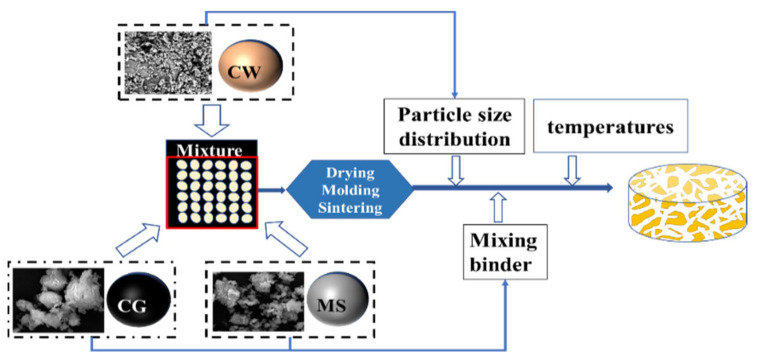
The preparation process of permeable bricks.

CG was purchased from Shendong mining area, Yulin City, Shaanxi Province. MS and CW were provided by the Inner Mongolia Shaanxi Coal Technology Co. Ltd., Xi’an, China. CG and MS were crushed and ground into powders below 0.1 mm using ball milling equipment after the dewatering process at 100 °C for 24 h. CW was crushed and ground into the distribution of 3.00–1.00 mm, which was sieved to the two aggregates’ gradation in the range of 3.00–2.00 and 2.00–1.00 mm by a standard sieving apparatus, acting as coarse and fine aggregates in permeable bricks, respectively. The mixing binder used MS and CG. Then, 10% of deionized water was added into the mixture with CW (80%), and the mixing binder (20%) lay for 24 h; this proportion guaranteed its properties based on our early experiments. Subsequently, the mixture was pressed into cylinders bodies (Φ64 × 24 mm) by a uniaxial hydraulic sampling machine (YES-2000 model Jinan, China) at 6 MPa, and the samples were dried in an oven at 105 °C for 2 h. The samples were sintered at 1125 °C, 1150 °C, 1175 °C, 1200, and 1225 °C, maintaining for 1 h in an electrical gradient furnace (GR 1300/13S, Nabertherm, Germany) at a heating rate 5 °C/min in air atmosphere. The bricks cooled to room temperature in the furnace. Table 1 shows the composition design of raw materials. The process flow is shown in Figure 1.

Elemental analysis was characterized using a Spectro Midex X-ray fluorescence analyzer. The mineralogical compositions of specimens were obtained using a XRD-6100 X-ray diffraction (XRD) analyzer under the following conditions: 40 Kv voltage, 40 mA current, and Cu Kα radiation (λ = 1.5406 Å). The microstructure and surface of the permeable bricks were measured using the VEGA II XMU afield-emission scanning electron microscopy (SEM) and optical camera.

According to the Chinese standard GB/T 25993-2010 (permeable paving bricks and permeable paving flags) [10,16], the permeability coefficient (Kt cm/s) is shown in Equation (1):

(1) $$K_{t}=\mathrm{Qd}/\left(\mathrm{AHt}\right)$$

where Q (mL) is the volume of water that flowed out. H (cm) and d (cm) represent the water level difference and the width of the permeable bricks, respectively. A (cm^2^) is the surface area of the sample measuring with a vernier caliper. T(s) is 5 min of scheduled time. Using waterproof glue around the sides of sample ensured the water flow from the upper surface to undersurface.

Based on the Archimedes principle [15], the apparent porosity is measured, which is shown in Equation (2):

(2) $$\varepsilon =\left(M_{1}-M_{2}\right)/\left(M_{1}-M_{3}\right)$$

where M_1_ (g) and M_2_ (g) are the oven-dried weight and the fully submerged weight of the sample, respectively. With the boiling time of 1 h, and M_3_ being the fully impregnated weight while suspended in deionized water, ε (%) is the apparent porosity of the samples.

The results from the tests above are the average value of three measurements.

## 3. Results and Discussion

### 3.1. Phase Analysis of Samples

The chemical composition results are demonstrated in Table 2, and the CG comprised 61.39%, 23.76%, and 4.14% of SiO_2_, Al_2_O_3_, and Fe_2_O_3_, respectively. Meanwhile, the CaO and Al_2_O_3_ reaching up to 56.08% are the main chemical compositions of MS. Therefore, the mixture of CG and MS with the content of SiO_2_, Al_2_O_3_, and CaO could serve as the mixing binder.

**Figure 2:**
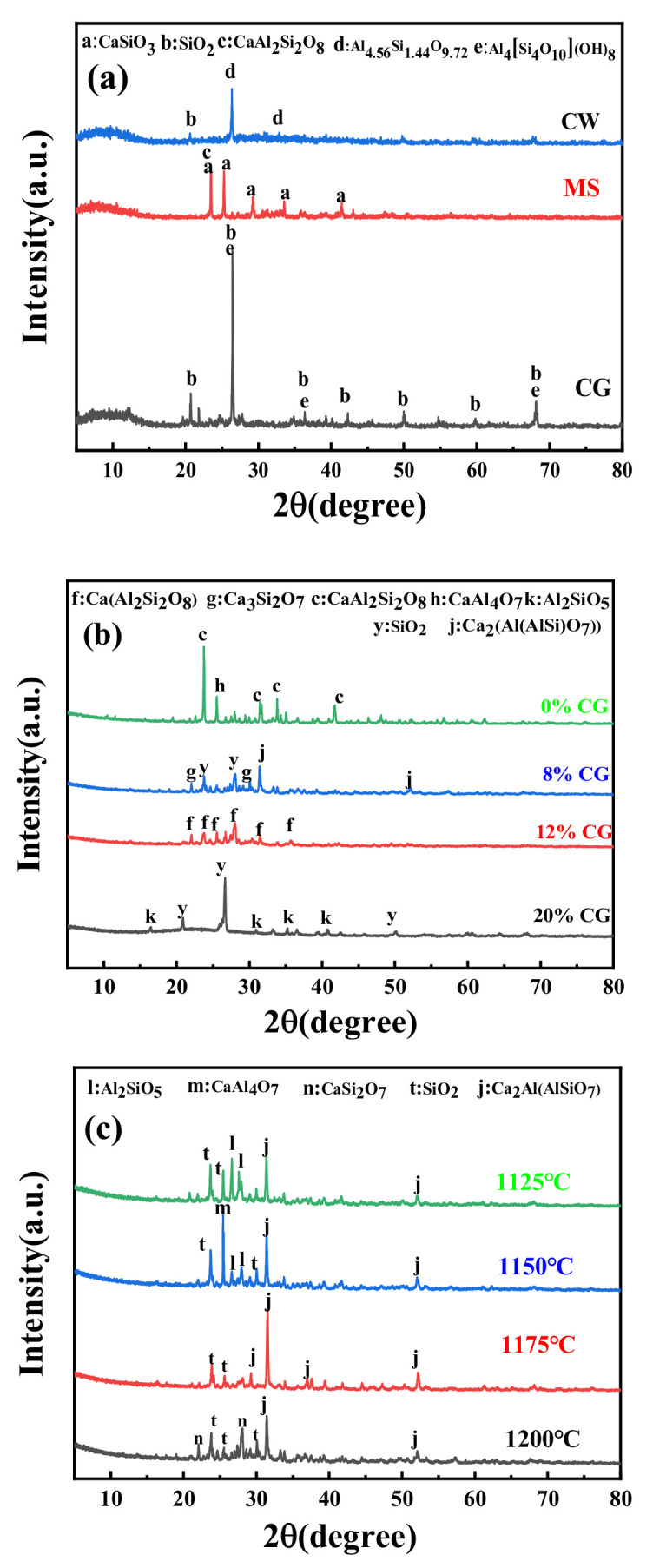
XRD pattern of samples: (**a**) raw materials; (**b**) mixing binder with different CG content sintered at 1200 °C; and (**c**) samples under different sintering temperatures with 8% CG in mixing binder.

The X-ray diffraction analysis spectra of raw materials are depicted in Figure 2a. The CG mainly consisted of quartz (SiO_2_, JCPDS Card 87–1780) and kaolinite (Al_4_[Si_4_O_10_](OH)_8_, JCPDS Card 78–2109) phases. The mineral phase of MS was composed of wollastonite (CaSiO_3_, JCPDS Card 72–2284) and dmisterinbergite (CaAl_2_Si_2_O_8_, JCPDS Card 74–0814) phases. The spectra of CW present mullite (Al_4.56_Si_1.44_O_9.72_, JCPDS Card 79–1458) and quartz phases. In addition, previously published papers [13] indicate that the presence of quartz and kaolinite of CG would lead to the dehydration reaction below 600 °C sintering temperature, and the quartz partial melting and glass formation when sintering temperature is more than 1100 °C. In addition, the naturally cooled magnesium slag scarcely contained the high activity β-C_2_S, and it rapidly became the low activity γ-C_2_S with cooling [18]. Generally, the intensities of the diffraction peak of CG were the highest compared with other materials. The XRD pattern analyses of samples sintered at 1200 °C are shown in Figure 2b. The mineral phases of samples mixed with CG and MS were complex, and their intensities of characteristic diffraction peaks were lower. Anorthite (Ca(Al_2_Si_2_O_8_) JCPDS Card 89–1461) and silicon oxide (SiO_2_ JCPDS Card 85–1780) were the predominant minerals of the sample with 12% CG. In addition, rankinite (Ca_3_Si_2_O_7_ JCPDS Card 73–0623) and gehlenite (Ca_2_(Al(AlSi)O_7_)) JCPDS Card 74–1607) phases were also evident in the sample with 8% CG, owing to the CG with SiO_2_ and Al_2_O_3_ and MS contained minerals with CaO. Therefore, the introduction of Ca contributed to the generation of the more liquid phases, decreasing the intensities of diffraction peaks. In addition, the content of liquid phases would influence the properties in many respects [24]. To further study the transformation mechanism of mineral phases, XRD was used to detect the composition of the sample with 8% CG at the sintering temperatures in the range of 1125–1200 °C. The results are demonstrated in Figure 2c. The gehlenite and silicon oxide (SiO_2_ JCPDS Card 85–1559) phases existed as the most stable phases in the whole range of sintering temperatures. Gehlenite was the highest peak of intensities of diffraction peaks, which were of great importance in the preparation of glass-ceramic [26]. In addition, the phases of rankinite and calcium aluminum oxide (CaAl_4_O_7_ JCPDS Card 74–1467) both transformed to gehlenite and silicon oxide with the sintering temperatures over 1150 °C. Besides, rankinite (CaSi_2_O_7_ JCPDS Card 76–0623) phases appeared in the patterns at a sintering temperature of 1200 °C. Comparing the peak intensities of samples sintered in the range of 1125–1200 °C, it is observed that the intensity of crystallinity was lowest at 1200 °C. The number of peaks presented in a rising tendency, with sintering temperatures decreasing from 1175 to 1125 °C, which could be attributed to the crystal melted and generation of liquid phases, and insufficient liquid phases resulted in the lower connection in the microstructure. In contrast, excessive liquid phases would have the opposite affect [27]. Macroscopically, the rankinite remained in samples and the increasing sintering temperature enhanced the formation of silicon oxide with an amorphous.

### 3.2. Microstructure of the Permeable Bricks

**Figure 3:**
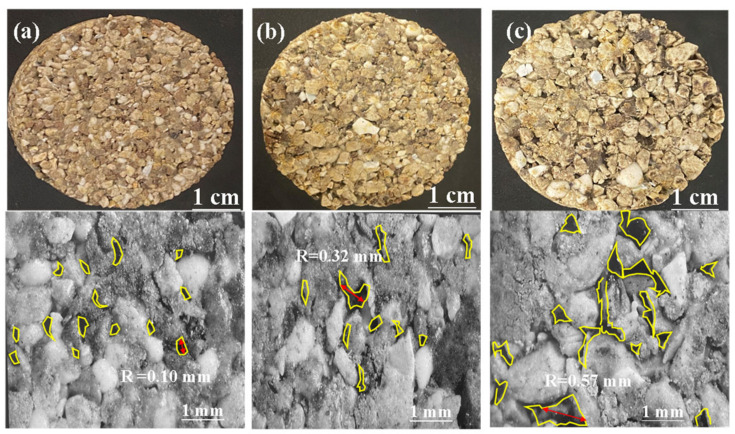
Optical images of the sample at 1200 °C with different content of coarse aggregate: (**a**) 0%; (**b**) 50%; and (**c**) 100%.

The internal structures of samples with various aggregate gradation are totally different, in that the skeleton of the sample using a high content of coarse aggregates was unstable, causing high permeability and low mechanical strength. The surfaces of permeable bricks are shown in Figure 3 with the different gradations of CW. It was apparent that the average size of pores rapidly expanded with the increase in the content of coarse aggregates, leading to the enhancement in the average size of the main pores over 200 μm. On the contrary, the sub-pores below 200 μm were dominated by fine aggregates. According to the Darcy law, the seepage coefficient of water passing through porous media per unit time is inversely proportional to the length of the percolation path [28]. However, superabundant introductions of coarse aggregates were bound to impair the mechanical property significantly for its unstable connection and high porosity in structure. By contrast, the fine aggregates resulted in better compressive strength, preventing the water permeation through the samples. Meanwhile, the fine aggregate has a larger specific surface area, which was more easily melted than coarser.

**Figure 4:**
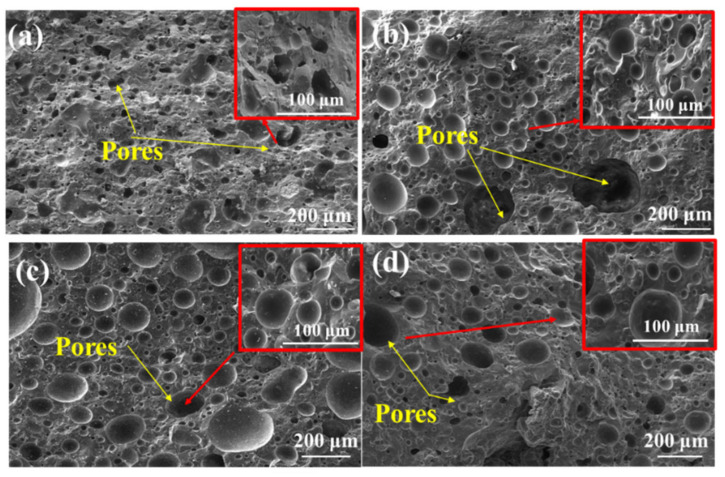
SEM morphologies of samples sintered at 1200 °C with different CG contents: (**a**) 0%; (**b**) 8%; (**c**) 12%; and (**d**) 20%.

SEM photographs in Figure 4 show the microstructure of samples with different ratios of CG in mixing binder at 1200 °C. The pores underwent variation significantly with the CG increasing content. It can be seen that the main pores existed in the samples with 8% CG, indicating that the remarkable shrinkage of mineral phases increased the number of pores. Meanwhile, the brick with 0% CG was obviously discontinuous and vulnerable. It could be inferred that MS led to a decrease in pores size and a loose crumb in the structure. Meanwhile, the SiO_2_ and Al_2_O_3_ introduced by CG were essential for the decisive influence on the strength of the brick [29]. According to the results, the mixing binders affected the pores by the meting phases, in that sample with 8% CG in mixing binder created the largest average size of pores, which indicated that the shrinkage of mixing binder was most obvious in the ratio.

**Figure 5:**
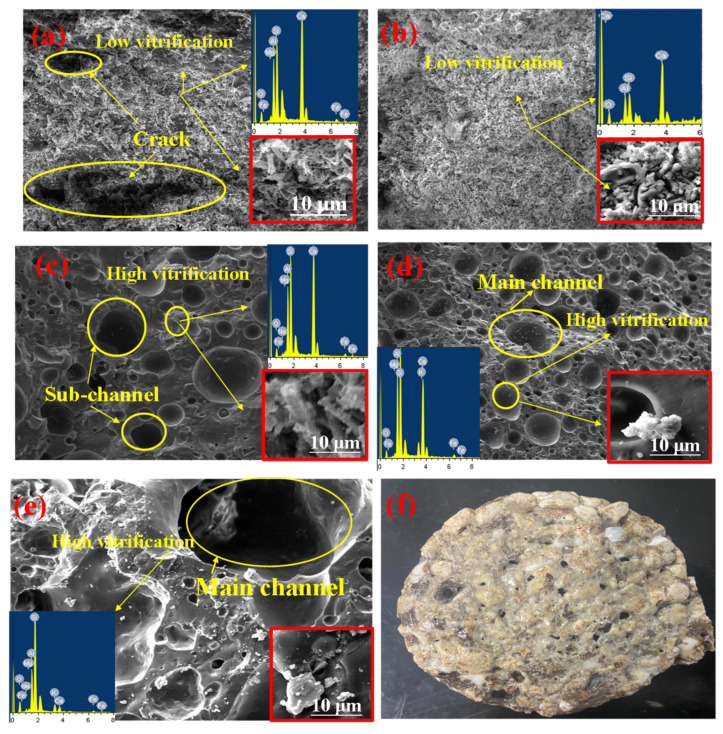
SEM photographs of samples at different temperatures: (**a**) 1125 °C; (**b**) 1150 °C; (**c**) 1175 °C; (**d**) 1200 °C; (**e**) 1225 °C; and (**f**) the brick sintered at 1225 °C.

To further study the relationship of microstructure and properties, the micrograph of the samples sintered at different sintering temperatures in the range of 1125–1225 °C is shown in Figure 5 with 80% of aggregates (%2.80–2.00 mm/%2.00–0.90 mm = 3:1) and 20% mixing binder (CG%/MS% = 2:3). It can be found that the microstructure was distinct and porous with the increased sintering temperature; at the same magnification of scanning electron microscopy, the shrinkage in melted liquid phases obviously was presented. The messy structure with a rough inner surface and acicular crystals developed at 1125 °C, indicating it failed to form the densification in the ceramic framework. With the increase in sintering temperatures, sufficient liquid phases were yielded, reinforcing the connection in the microstructure. Notably, the pores expanded significantly as the sintering temperatures increased to 1225 °C. The aggregates reached the melting points to which the internal structure expanded significantly in Figure 5f.

The EDS results also demonstrated in Table 3 that the content of Si and Al increased gradually, and the content of O presented a contrary tendency with the increase in sintering temperatures. There also existed elements like Fe, Mg, and Na in the sample. With the growth in sintering temperatures, the system was basically constructed. There were no traces of microscopic pores below 1175 °C, and the microstructure in 1200 °C was distinct and well-developed. By contrast, the sample showed the gular shape with sintering temperatures over 1150 °C, leading to the main pores dominating the permeability in the system.

### 3.3. Influence of Aggregate Gradation of Particle Size on Sample Properties

In this work, the influence of different aggregate gradation was detected, which used 80% of waste ceramic materials material and 20% of mixing binders (CG%:MS% = 1:1) at 1200 °C for 1 h. The theory of fractal seepage supposed that the permeability is not just determined by apparent porosity, and the factors of the radius of pores also played an important role. According to the Hagen–Poiseulle equation [30,31],

$$q\left(r\right)=\frac{\pi }{128\mu }\frac{\Delta P}{L_{f}}r^{4}$$

**Figure 6:**
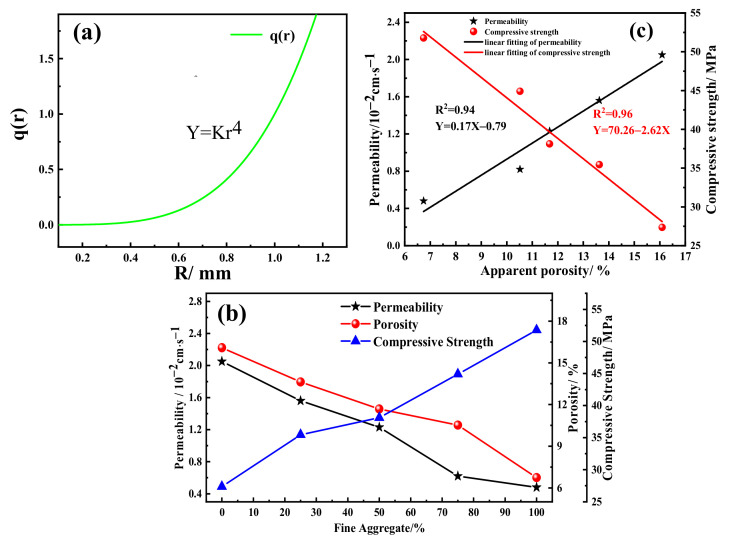
(**a**) The influence of radius on the velocity of water flow; (**b**) the trend f permeability, apparent porosity, and compressive strength with different fine aggregate content; and (**c**) fitting curves of compressive strength and permeability with apparent porosity.

The large calculation of the equation reflected permeability. q(r) is on behalf of the flow rate of a single capillary. L_f_ is the tortuous length of the capillary; μ is the fluid viscosity; ΔP is the pressure difference on capillary, and r represented the radius of pores. According to the permeable experimental, the certain hydraulic pressure on the bricks guaranteed ΔP as the constant. Figure 6a demonstrates that the radius significantly affected permeability. the L_f_ could be ignored when the r increased rapidly, owing to the r affecting the q(r) in quadruplicate. Figure 6b shows that, with the increase in the content of coarse aggregate, the permeability of samples ranged from 2.05 × 10^−2^ to 0.48 × 10^−2^ cm/s, and the compressive strength increased from 27.37 to 51.87 MPa. The apparent porosity acquired the maximum value of 16.09% with 100% of coarse aggregates. The linear fitting curves in Figure 6c demonstrate clearly that the permeability(y) and apparent porosity(x) were expressed as y = −0.79 + 0.17 x with R^2^ = 0.94. Meanwhile, the compressive strength(y) and porosity(x) were approximated as y = −2.62 x + 70.26 with R^2^ = 0.96. The results indicated that permeability and compressive strength both have a high correlation relationship with apparent porosity. The results generally conformed to the linear model [32].

The samples using a high content of fine aggregates were likely to fill pores in volume, inevitably reducing the porosity [33]. It was acknowledged that the main pores enhanced the permeability significantly, while the sub-pores improved the mechanical property significantly. To obtain the appropriate performance of permeable brick, the coarse and fine aggregates were selected as 60% and 20%.

### 3.4. Influence of Mixing Binder Composition on Sample Properties

**Figure 7:**
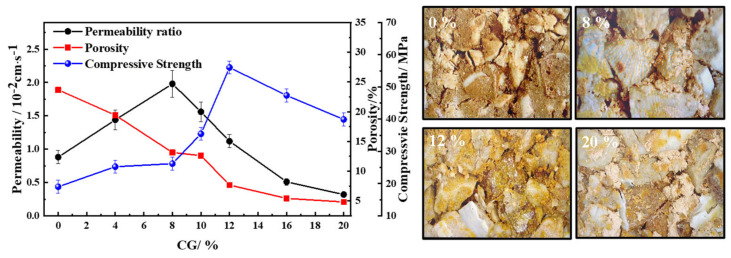
The trend of permeability coefficient, apparent porosity, and compressive strength with different CG contents at 1200 °C.

The affection of mixing binder composition on the properties was discussed. With 60% coarse aggregate, 20% fine aggregate and 20% of mixing binders sintered at 1200 °C for 1 h. Figure 7 shows the compressive strength and permeability, and that the maximum compressive strength was 56.04 MPa at the ratio of 12% CG. Meanwhile, the permeability coefficient acquired the maximum of 1.98 × 10^−2^ cm/s with 8% of CG, which is higher than the Chinese national standard of 0.01 cm/s [25,27]. The apparent porosity increased from 4.83 to 20.68% with the decreasing content of CG. The results could be attributed to the fact that the mixing binder with 10% CG introduced the suitable content of Cao, SiO_2_, and Al_2_O_3_, improving the properties [34]. By contrast, the mixing binder using single CG or MS demonstrated low compressive strength and water permeability. From the high intensities of diffraction peaks in XRD, the mixing binder could not form the tight cohesive force, leading to the low mechanical strength. It could be seen in Figure 4 that the microstructure mainly consisted of sub-pores, which subsequently prevented the water from moving through the samples. The different ratios of mixing binders influenced the multi-pore permeable system, generating different kinds and quantities of melted liquid phase in microstructures. According to Figure 7, the compressive strength of the mixing binder with 12% CG was obviously stronger, which was attributed to the content of CG and MS producing the highest cohesive strength. The porous microstructure in Figure 4b facilitated the highest permeability with the mixing binder using 8% CG. The mixing binder using 10% CG generated moderate liquid phases, causing the permeable bricks to have 35.5 MPa and 1.56 × 10^−2^ cm/s permeability.

### 3.5. Effect of Sintering Temperature on Sample Properties

**Figure 8:**
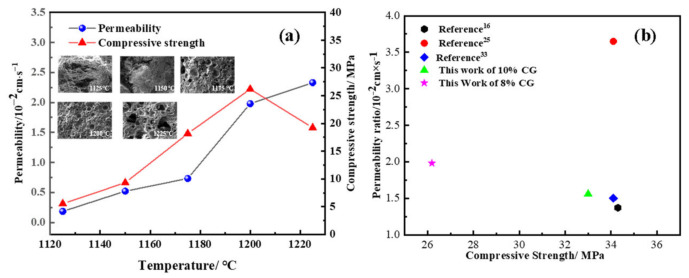
(**a**) The trend of permeability and compressive strength in the sample sintered at different temperatures; (**b**) the comparison of this work with bricks in other references.

The results were exhibited in Figure 8, discussing the influence of sintering temperatures on compressive strength and permeability. The samples consisted of 60% coarse aggregate, 20% fine aggregate, 8% coal gangue and 12% magnesium slag at 1125, 1150, 1175, 1200, and 1225 °C sintering temperatures for 1 h. Figure 8a demonstrates the trend of water permeability and compressive strength. Notably, the properties improved significantly with temperatures over 1150 °C, and the maximum of permeability coefficient appeared at 1225 °C. However, the compressive strength rapidly decreased with sintering temperatures over 1200 °C. This phenomenon could be explained in that the sintering temperatures increasing to 1200 °C contributed to the vitrification of the melted liquid phase, generating an excellent coherence force of liquid phases in the microstructure [35]. The shrinkage of the mineral phase generated multiple connected channels and tight conjoined points in the microstructure. However, when the sintering temperatures exceeded 1200 °C, the melted aggregates led to the collapse of the internal microstructures, where the high connected channels among the samples resulted in high permeability and low mechanical strength. In the system, the mechanical strength increased in the range of 1125–1200 °C, which was dominated by the cohesive force of mixing binders. When the aggregates melted, more main pores presented, leading to the significantly decrease in compressive strength at 1225 °C. As shown in Figure 8b, it shows a great combination property compared with other references [16,25,33].

## 4. Conclusions

All solid-waste-based permeable bricks with waste ceramic materials, coal gangue, and magnesium slag were synthesized via pressing-sintering process. We could draw the following conclusions:

1. The porous channel over 200 μm was mainly influenced by the coarse aggregate of waste ceramic materials, which determines the water permeability. Pore structures below 200 μm were mainly regulated by fine aggregate and mixed binder, and play an important role in mechanical properties.
2. The all-solid-waste-based permeable bricks were prepared by pressing at 6 MPa and sintering at 1200 °C with 60% coarse aggregate, 20% fine aggregate, 10% coal gangue, and 10% magnesium slag. It showed the best properties and its water permeability, compressive strength, and apparent porosity were 1.56 × 10^−2^ cm/s, 35.45 MPa, and 13.15%, respectively. Excellent performance of the all-solid-waste-based permeable bricks was attributed to the porous microstructure of waste ceramic materials and the interaction of liquid phases of coal gangue and magnesium slag.
3. The research broadened the application of coal gangue and magnesium slag in the selection of pavement materials. At the same time, it is possible to realize the large-scale and resource utilization of coal gangue and magnesium slag solid waste, which plays a positive role in ecological environment protection, but the engineering application still needs to be demonstrated by a large number of experiments.

## Figures and Tables

**Table 1:** The mix proportion of permeable bricks.

Sample	Fine Aggregate/%	Coarse Aggregate/%	CG/%	MS/%	°C
A-1	80	0	10	10	1200
A-2	60	20	10	10	1200
A-3	40	40	10	10	1200
A-4	20	60	10	10	1200
A-5	0	80	10	10	1200
B-1	60	20	0	20	1200
B-2	60	20	4	16	1200
B-3	60	20	8	12	1200
B-4	60	20	10	10	1200
B-5	60	20	12	8	1200
B-6	60	20	16	4	1200
B-7	60	20	20	0	1200
C-1	60	20	8	12	1125
C-2	60	20	8	12	1150
C-3	60	20	8	12	1175
C-4	60	20	8	12	1200
C-5	60	20	8	12	1225

**Table 2:** The chemical composition of raw materials (mass fraction, %).

Samples	CaO	MgO	SiO_2_	Al_2_O_3_	Fe_2_O_3_	TiO_2_	K_2_O	Na_2_O	Loss on Ignition
MS	39.57	1.18	6.64	16.51	1.77	0.67	0.72	0.46	17.03
CG	0.82	1.57	61.39	23.76	4.14	0.79	2.82	1.62	2.11
CW	1.17	0.64	39.10	12.2	1.28	0.34	2.18	2.09	40.02

**Table 3:** EDS analysis of samples at different sintering temperatures: (a) 1125 °C; (b) 1150 °C; (c) 1175 °C; (d) 1200 °C; and (e) 1225 °C.

Elements	O	Si	Al	Ca	Fe	Mg	Na	TOTAL
(a)	39.49	10.73	10.94	38.84	0	0	0	100
(b)	24.04	18.87	12.94	40.30	2.71	1.13	0	100
(c)	18.42	23.50	14.27	39.67	2.69	0.79	0.67	100
(d)	19.84	26.27	14.62	31.53	4.94	1.63	0	100
(e)	16.00	27.51	17.37	30.89	2.90	3.48	0.5	100

## Data Availability

The data used to support the findings of this study are available from the corresponding author upon request.
